# Retraction Note: Atovaquone inhibits colorectal cancer metastasis by regulating PDGFRβ/NF-κB signaling pathway

**DOI:** 10.1186/s12885-026-16535-9

**Published:** 2026-07-20

**Authors:** Bin Liu, Xin Zheng, Jiajun Li, Peng Yao, Peng Guo, Wei Liu, Gaoping Zhao

**Affiliations:** 1 Department of General Surgery, The Second Affiliated Hospital of Chengdu Medical College, National Nuclear Corporation 416 Hospital, Chengdu, Sichuan 610051 China; 2 Department of Gastrointestinal Surgery, Sichuan Academy of Medical Sciences, Sichuan Provincial People’s Hospital, Chengdu, Sichuan 610072 China; 3 Department of Nephrology, The Second Affiliated Hospital of Chengdu Medical College, National Nuclear Corporation 416 Hospital, Chengdu, Sichuan 610051 China; 4 Chengdu Medical College, Chengdu, Sichuan 610500 China; 5 Department of Hepatobiliary Surgery, The First Affiliated Hospital of Chongqing Medical University, Chongqing, China

**Retraction Note: BMC Cancer** **23, 1070** **(2023)**

**https://doi.org/10.1186/s12885-023-11585-9**

The Editors have retracted this article. After publication, concerns were raised about some of the data in this Article. Specifically, similarities have been detected.

• between panels within Fig. 1.

• between Figs. 1 and 3.

• between Figs. 1 and 4.

• between Figs. 2 and 4.

• between Figs. 3 and 4.

• between panels within Fig. 4.

The Authors were unable to provide a sufficient response to concerns. The Editors have therefore lost confidence in the data and conclusions of this Article.

Author Bin Liu has stated on behalf of all authors that all authors disagree with this retraction.

